# Genome-Wide Analysis Reveals the Roles of *FAR1/FHY3* Genes in *Fragaria* × *ananassa* Under Abiotic/Biotic Stresses and Highlights Their Potential Functions in Anthocyanin Biosynthesis During Fruit Development

**DOI:** 10.3390/ijms27125479

**Published:** 2026-06-17

**Authors:** Ruoxue Ren, Yujia Xu, Yueqi Cheng, Qiuping Li, Wona Ding, Lan Shen, Lili Chen

**Affiliations:** 1 Ningbo Key Laboratory of Agricultural Germplasm Resources Mining and Environmental Regulation, College of Science and Technology, Ningbo University, Ningbo 315300, China; 13989619836@163.com (R.R.); xuyujia@nbu.edu.cn (Y.X.); chengyueqi@nbu.edu.cn (Y.C.); liqiuping2@nbu.edu.cn (Q.L.); dingwona@nbu.edu.cn (W.D.); 2 Ningbo Academy of Agricultural Sciences, Ningbo 315040, China

**Keywords:** *Fragaria* × *ananassa*, *FAR1/FHY3* transcription factor, anthocyanin biosynthesis, fruit development, multiple stresses

## Abstract

*FAR1/FHY3* transcription factors are key regulators of plant growth and development, but their identification and functions in strawberries (*Fragaria* × *ananassa*) remain largely unexplored. In this study, 47 *FaFAR1/FHY3* genes in cultivated strawberries were systematically identified, which were categorized into six subfamilies and randomly distributed across 15 chromosomes, with segmental duplication as the main driver of the expansion of this gene family. Integration of phylogenetic relationships, gene structure, and conserved motif composition uncovered distinct divergences among the subfamilies. A *cis*-acting element analysis of promoters and gene expression profiles showed that these genes responded to various abiotic and biotic stresses, phytohormones, and far-red light signals, with *FaFAR1-7* and *FaFAR1-44* strongly responding to multiple stresses, including temperature, drought, and pathogen infection. Additionally, *FaFAR1-12* and *FaFAR1-18* exhibited positive correlations with anthocyanin accumulation and the expression of key anthocyanin biosynthesis genes during fruit development. Dual-luciferase reporter assays further confirmed that FaFAR1-12 and FaFAR1-18 significantly activated the promoters of key structural genes related to anthocyanin biosynthesis, indicating that these two TFs exert vital regulatory functions in anthocyanin accumulation during strawberry fruit development. This study comprehensively identifies and characterizes the *FaFAR1/FHY3* genes in cultivated strawberries, laying a foundation for their functional analysis and for screening out the key regulatory genes for strawberry fruit quality improvement.

## 1. Introduction

*FAR1* (far-red impaired Response 1)/*FHY3* (far-red elongated hypocotyl 3), new transcription factors (TFs) derived from the mutator transposase, are structurally characterized by an N-terminal C2H2-type zinc finger-chelating DNA-binding domain, a central putative core transposase domain, and a highly conserved C-terminal SWIM domain [1]. The versatile roles of FAR1/FHY3 proteins are highlighted by their features, underscoring their multifaceted functions in plants. Recently, multiple studies have revealed the crucial role of *FAR1/FHY3* in plant growth and developmental processes, including light signal transduction [2,3], plant shoot branching and flowering [4,5], plant chloroplast division and chlorophyll biosynthesis [6,7], circadian clock regulation [8,9], ABA signaling [10,11], and reactive oxygen species (ROS) homeostasis [12].

Plants have developed extremely sophisticated light-sensing systems to regulate their growth and development [13]. Phytochromes are primarily photoreceptors that sense red and far-red (FR) light [13]. *FAR1/FHY3* TFs are crucial components of phytochrome signaling transduction pathways [8,14]. *FAR1/FHY3* genes act as vital initiators under continuous FR light irradiation for the de-etiolation of *Arabidopsis thaliana* L. seedlings, a response that is exclusively sensed by phytochrome A (phyA) [14]. The above processes reflect the redundant functions of *FHY3* and *FAR1*, but the dominant function of *FHY3* can only be partly replaced by *FAR1* [15]. Moreover, the proteins encoded by *FAR1/FHY3* interact with photo-activated phyA and mediate its nucleus import, thereby triggering downstream transcriptional programs [8]. Furthermore, FAR1/FHY3 proteins are essential for phytochrome B (phyB)-mediated maintenance of the expression of *ELF4*, which acts as a core circadian clock integrating light and temperature to modulate circadian rhythms, flowering time, and stress tolerance in *A. thaliana* [16].

*FAR1/FHY3* directly bind to the promoter of *COP1* gene and activate its transcription under UV-B light [10]. *ELF4* expression is upregulated by *FAR1/FHY3* through coupling with miR156-*SPL* module-mediated light signaling, affecting the flowering process [5]. Plant starch synthesis is also regulated by *FAR1/FHY3* via activating the expression of *ISA2* gene [6]. Through interacting with *SPL9/SPL15*, *FAR1/FHY3* TFs also affect plant branching [17]. Numerous putative downstream targets genes of *FAR1/FHY3* TFs are found in *A. thaliana*, signifying the wide-ranging biological functions of these TFs, to be explored further [18]. Furthermore, *FAR1/FHY3* TFs actively participate in plant responses to biotic and abiotic stresses, including plant–pathogen interactions [7], low/high temperatures [19], drought, and salt stress [20], which are of great significance for elucidating plant adaptation to the external environment at the molecular level. Briefly, the functional diversity of *FAR1/FHY3* TFs renders them indispensable for dissecting the regulatory mechanisms underlying plant signaling networks. Currently, *FAR1/FHY3* genes have been identified in many plants, such as *A. thaliana* [21], maize [22], tea [23], and cucumber [24]. However, the identification and characterization of *FAR1/FHY3* TFs have not been reported in strawberries.

Strawberry (*Fragaria* × *ananassa*) fruits are deeply loved by consumers for their bright color, delicious taste, and rich nutrition [25]. According to the FAO, its production ranks first among all small berry fruits, making it an important fruit worldwide [26]. It is well known that strawberry production is challenged by various biotic stresses (e.g., powdery mildew, gray mold, and root rot) and abiotic stresses (e.g., low temperature, low light intensity, drought, and salt stress) [27]. In addition, anthocyanins are key determinants of strawberry fruit quality. Numerous studies have demonstrated that *FAR1/FHY3* and other TFs play essential regulatory roles in anthocyanin biosynthesis in plant fruits [28,29,30]. However, the in-depth understanding of *FAR1/FHY3* TFs in strawberries is poor. Given the importance of strawberries and the challenges posed by multiple stresses, research on the *FaFAR1/FHY3* genes in strawberries is crucial.

In this study, we systematically identified the *FaFAR1/FHY3* genes in *F. ananassa* and analyzed their chromosomal localization, phylogenetic relationships, conserved motifs, gene structures, and synteny. Moreover, the *cis*-regulatory elements of the *FaFAR1/FHY3* genes were predicted, and their expression patterns under biotic and abiotic stresses, phytohormone treatments, FR light signaling, and during strawberry fruit development were analyzed. Furthermore, the putative functions of the core *FaFAR1/FHY3* genes in anthocyanin biosynthesis during strawberry fruit development were focused on. The results elucidate the evolutionary traits and functional diversity of *FaFAR1/FHY3* in strawberries and highlight their potential roles in regulating anthocyanin biosynthesis in strawberry fruit.

## 2. Results

### 2.1. Identification, Chromosomal Distribution, and Collinearity Analysis of FaFAR1/FHY3 Genes

**Figure 1:**
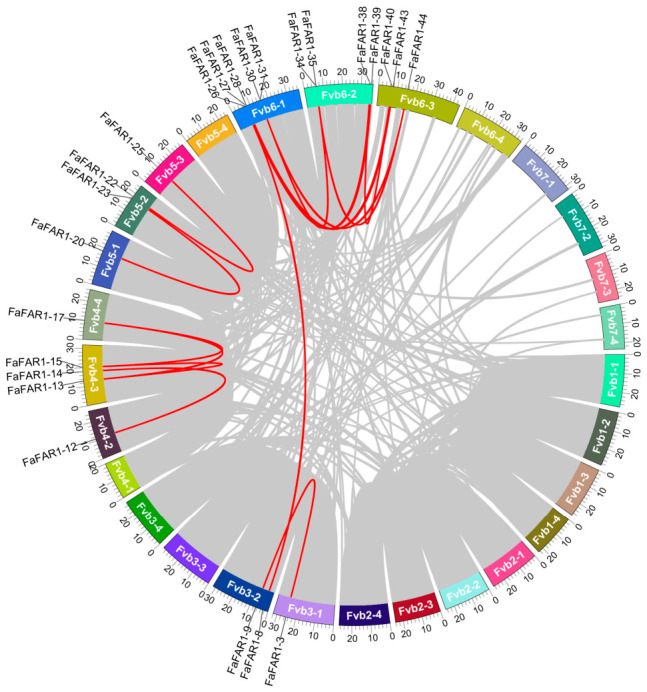
Synteny analysis of *FaFAR1/FHY3* genes. Red lines represent segmentally duplicated gene pairs.

Based on the conserved domain, 47 *FaFAR1/FHY3* genes were confirmed in *F. ananassa* (Table 1) and sequentially named *FaFAR1-1* to *FaFAR1-47* according to their positions on the chromosomes (Figure S1). The 47 *FaFAR1/FHY3* genes were randomly distributed across 15 chromosomes, with the largest number present on chromosome Fvb6-1. Only one gene was found on chromosomes Fvb2-1, Fvb2-2, Fvb3-3, Fvb4-2, and Fvb7-4 (Figure S1). Furthermore, three tandem duplications occurred: one pair consisting of *FaFAR1-30* and *FaFAR1-31* on chromosome Fvb6-1, and two clusters containing three genes each on chromosomes Fvb6-2 (*FaFAR1-34*, *FaFAR1-35*, and FaFAR1-36) and Fvb6-3 (FaFAR1-41, FaFAR1-42, and *FaFAR1-43*) (Figure S1). Additionally, 17 pairs of duplicated segments were identified on 11 chromosomes (Figure 1 and Table S1). An orthologous gene analysis revealed 20, 16, 0, 38, 34, 36, 0, and 35 orthologous gene pairs between strawberries and the other eight species, including *Solanum lycopersicum* L., *A. thaliana*, *Oryza sativa* L., *Pyrus communis* L., *Rosa chinensis* Jacq., *Prunus persica* L., *Zea mays* L., *and Rubus fruticosus* L. (Figure S2).

The length of FaFAR1/FHY3 proteins varied from 524 aa (FaFAR1-20) to 941aa (FaFAR1-4), which is highly consistent with the variation in molecular weight (Table 1). The isoelectric points of FaFAR1/FHY3 proteins ranged from 5.26 (FaFAR1-32) to 9.10 (FaFAR1-37), with 24 acidic proteins (pI < 7) and 23 basic proteins. The instability index ranged from 38.98 (FaFAR1-7) to 53.32 (FaFAR1-41). Five proteins had values below 40 and were classified as stable, whereas the remaining proteins had values above 40 and were thus unstable (Table 1). All FaFAR1/FHY3 proteins were hydrophilic due to their negative grand average of hydropathicity values (Table 1).

### 2.2. Motif, Domain, and Gene Structure Analysis of FaFAR1/FHY3 Genes

**Figure 2:**
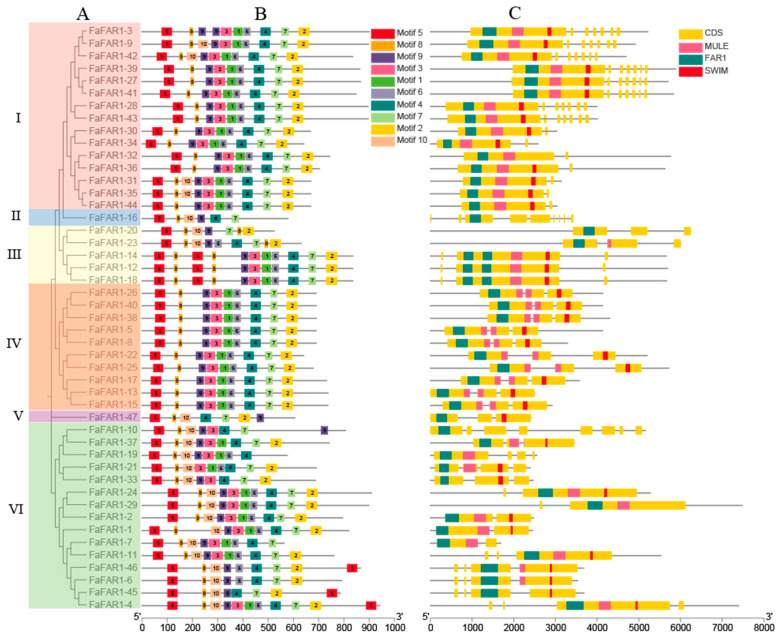
Evolutionary characteristics (**A**), motifs (**B**), and structure and domain (**C**) analysis of *FaFAR1/FHY3* genes.

The sequence alignment revealed that the FaFAR1/FHY3 proteins were highly conserved and were classified into six subfamilies by a phylogenetic analysis (Figure 2A). To further characterize the FaFAR1/FHY3 proteins, 10 conserved motifs were identified (Figure S3). Evolutionary conservation of all FaFAR1/FHY3 proteins was indicated by motif 5, 7, 8, and 9 (Figure 2B). Identical or highly similar motif compositions were observed in genes belonging to the same subfamily (Figure 2B). Except for three proteins (FaFAR1-20, FaFAR1-10, and FaFAR1-16) harboring only the FAR1 domain and two proteins (FaFAR1-29 and FaFAR1-19) containing both the MULE and FAR1 domains, all the remaining FaFAR1/FHY3 proteins contained three conserved domains: MULE, FAR1, and SWIM (Figure 2C). The longest gene was *FaFAR1-29*, while *FaFAR1-7* was the shortest (Figure 2C). Meanwhile, eight genes had six introns, and the other genes had only one intron in Group I. The structure of *FaFAR1-6* in Group II was relatively complex, with the largest number of introns (nine). However, a relatively simple structure was observed in the Groups III–V genes, each with two introns. In addition, the number of introns of genes in Group VI ranged from one to seven (Figure 2A,C).

### 2.3. Phylogenetic Analysis of FAR1/FHY3 Proteins

**Figure 3:**
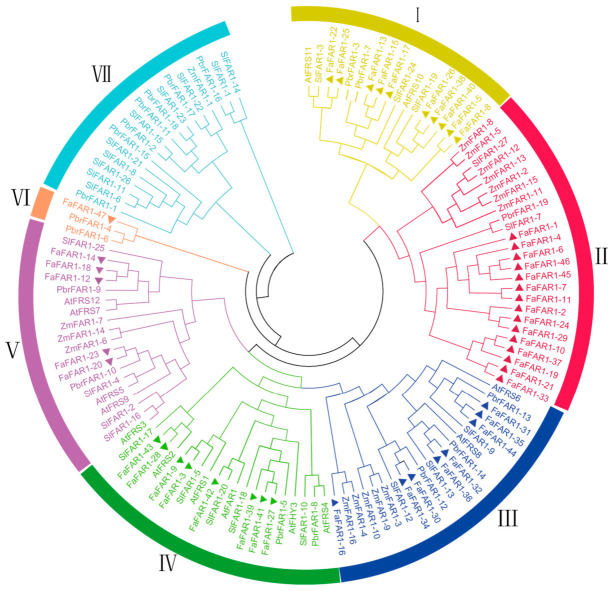
Genetic evolutionary relations of FAR1/FHY3 proteins in strawberries, pears, maize, tomatoes, and *Arabidopsis*. The triangle symbol represents the strawberry FaFAR1/FHY3 proteins. Different subfamilies are shown in different colors.

A phylogenetic tree was constructed with 123 protein sequences from *F. ananassa*, *S. lycopersicum*, *A. thaliana*, *O. sativa*, and *Pyrus bretschneideri* Rehd to characterize the genetic evolutionary relationships of FAR1/FHY3 proteins. The FAR1/FHY3 proteins were divided into seven major groups (Groups I–VII) (Figure 3). Groups I to VII contained 10, 15, 8, 8, 5, 1, and 0 FaFAR1/FHY3 proteins, respectively (Figure 3). In Group II, 15 FaFAR1/FHY3 proteins were closely clustered and clearly distinct from those in other plants, suggesting that these proteins have undergone a certain evolutionary divergence. The FaFAR1/FHY3 proteins in Groups II–V were scattered among those of other species, and most of them were closely clustered with the proteins from *A. thaliana*, *S. lycopersicum*, and *P. bretschneideri*, indicating the high conservation of these proteins and their close evolutionary relationships with these three species (Figure 3). However, FaFAR1-47 and two FAR1 proteins from *P. bretschneideri* were present in Group VI (Figure 3), further supporting the idea that FaFAR1/FHY3 proteins are evolutionarily closer to the dicotyledons, especially the species, in the *Rosaceae* family. These results are generally consistent with the interspecific collinearity analysis shown in Figure S2.

### 2.4. Cis-Acting Element Analysis of FaFAR1/FHY3 Promoters

**Figure 4:**
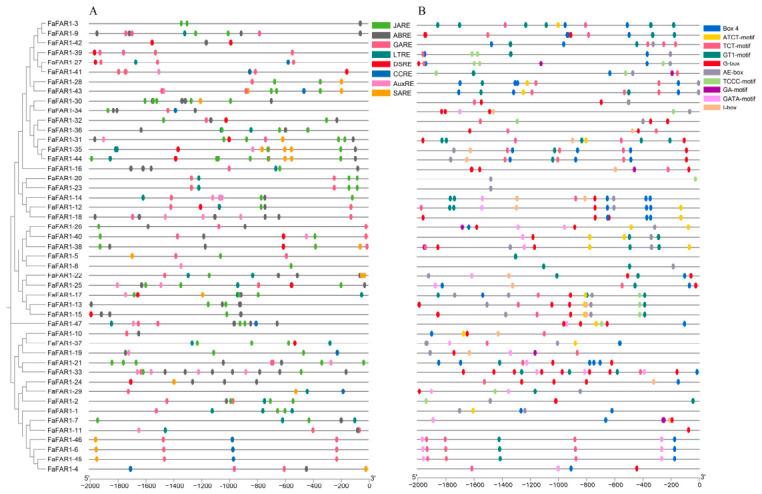
*Cis*-acting elements analysis of the promoters of *FaFAR1/FHY3* genes. (**A**) Elements associated with hormone response, growth and stress tolerance are presented. JARE: MeJA-responsive element; ABRE: abscisic acid-responsive element; GARE: gibberellin-responsive element; SARE: salicylic acid response element; LTRE: low-temperature response element; DSRE: defense and stress response element; CCRE: circadian control element; AuxRE: auxin-responsive element. (**B**) Light response elements are presented.

A *cis*-acting element prediction was performed on the 2000 bp upstream sequences of the *FaFAR1/FHY3* genes, identifying 827 *cis*-acting elements (Figure 4 and Table S2). The light-responsive elements (LREs) were found to be the largest elements, constituting 49.2% of all *cis*-acting elements, primarily including AE-box, G-box, I-box, Box 4, ATCT-motif, GATA-motif, GA-motif, GT1-motif, TCCC-motif, and TCT-motif (Figure 4B and Table S2). The second largest category included five types of hormone-responsive elements, such as 78 CGTCA-motifs and 78 TGACG-motifs linked with jasmonic acid (JA) responsiveness; 88 elements related to abscisic acid (ABA) responsiveness; 24 p-boxes, 28 GARE-motifs, and eight TATC-boxes involved in gibberellin (GA) responsiveness; and 26 TGA-elements associated with auxin responsiveness. Furthermore, 3.5% were low-temperature-responsive elements, and TC-rich repeats associated with defense and stress responsiveness represented 2.0%. Circadian control elements were the least abundant elements, with a total of 11, accounting for only 1.3% (Figure 4 and Table S2). These findings reveal that the *FaFAR1/FHY3* genes might play crucial roles in light, hormone, low-temperature, defense, and stress responses.

### 2.5. Expression Analysis of FaFAR1/FHY3 Genes Under Multiple Stressors

**Figure 5:**
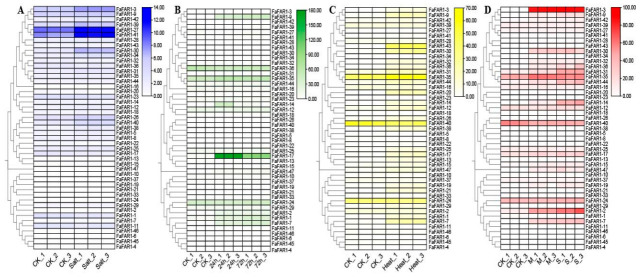
Expression analysis of *FaFAR1/FHY3* genes under abiotic stress. Blue (**A**), green (**B**), yellow (**C**), and red (**D**) indicate salinity, cold (4 °C), heat (40 °C), and drought stress, respectively. Results are from three biological replicates. Transcript abundance is normalized to transcripts per million (TPM) for gene expression analysis. M: mild drought; S: severe drought. Darker colors represent higher transcript abundance, while lighter colors represent lower expression levels.

The *cis*-acting element prediction revealed that *FaFAR1/FHY3* harbored numerous stress-responsive elements. Therefore, the responses of these genes to abiotic stress were further examined (Figure 5). The transcript levels of *FaFAR1-3*, *FaFAR1-27*, *FaFAR1-30*, and *FaFAR1-41* were significantly upregulated under salt stress (Figure 5A). Although most genes showed no obvious expression under cold stress, *FaFAR1-1*, *FaFAR1-7*, *FaFAR1-9*, and *FaFAR1-17* showed significantly increased expression (Figure 5B). *FaFAR1-17* at 24 h of cold treatment exhibited the highest expression, followed by a slight decrease at 72 h, but it remained significantly higher than that of the other genes (Figure 5B). Almost less than half of the *FaFAR1/FHY3* genes showed positive responses to heat stress, among which *FaFAR1-7*, *FaFAR1-30*, and *FaFAR1-43* showed the highest expression (Figure 5C). Drought stress was well responded to by about 45% of *FaFAR1/FHY3* genes. *FaFAR1-3* was well induced by mild and severe drought. Under mild drought, the transcript levels of *FaFAR1-2*, *FaFAR1-7*, *FaFAR1-31*, and *FaFAR1-44* showed higher levels than those of the control, and were further elevated under severe drought, indicating a gradual increase upon drought stress intensification (Figure 5D).

Regarding biotic stress, most *FaFAR1/FHY3* genes were barely expressed after inoculation with *Xanthomonas fragariae*, whereas *FaFAR1-7* showed consistently high expression. At 12 days post-inoculation (dpi), FaFAR1-9 was undetectable, but its expression level was obviously higher compared to that of the control, at 19 dpi (Figure S4A). After inoculation with *Colletotrichum acutatum*, no obvious expression was noticed in most of *FaFAR1/FHY3* genes except the upregulated *FaFAR1-35*, *FaFAR1-41*, and *FaFAR1-44* (Figure S4B). These results suggest that *FaFAR1-7* and *FaFAR1-44* actively responded to biotic and abiotic stresses.

### 2.6. Characterization of FaFAR1/FHY3 Genes Responses to Hormone Treatment

**Figure 6:**
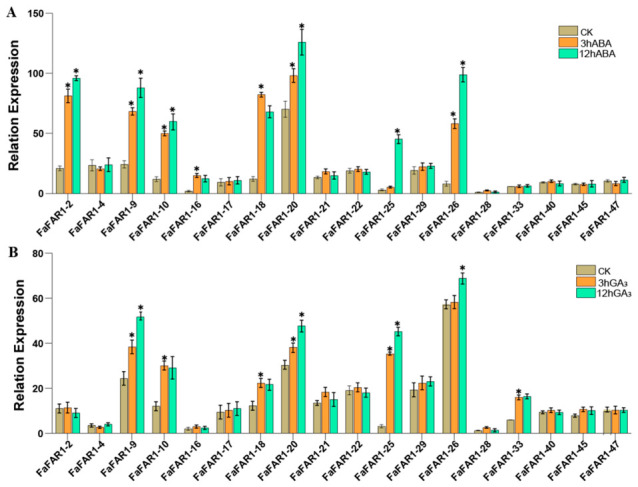
Expression patterns of *FaFAR1/FHY3* genes under ABA (**A**) and GA_3_ (**B**) hormone treatments. Significant differences are shown with an asterisk (*) at the *p* < 0.05 level.

Given that the *FaFAR1/FHY3* genes contain numerous *cis*-acting elements associated with hormone responses, the expression of the *FaFAR1/FHY3* genes in response to different hormones was analyzed. Genes such as *FaFAR1-2*, *FaFAR1-9*, *FaFAR1-10*, *FaFAR1-20*, and *FaFAR1-26* reached their peak values after 12 h of ABA treatment, which were significantly higher than those after a 3 h ABA treatment and CK (Figure 6A), signifying that such genes might function in ABA signaling. However, no significant changes were observed in the expression levels of *FaFAR1-4*, *FaFAR1-17*, *FaFAR1-21*, *FaFAR1-22*, *FaFAR1-28*, *FaFAR1-29*, *FaFAR1-33*, *FaFAR1-40*, *FaFAR1-45*, or *FaFAR1-47* after ABA treatment. Even a slight downregulation was observed for certain genes, indicating different responses of the *FaFAR1/FHY3* genes to ABA signaling.

Diverse expression patterns were observed in the *FaFAR1/FHY3* genes after gibberellin (GA_3_) treatment. The highest expression level was observed in *FaFAR1-26* after 12 h GA_3_ treatment. Considerably upregulated expression levels were noticed in *FaFAR1-9*, *FaFAR1-20*, *FaFAR1-25*, and *FaFAR1-26* after 12 h GA_3_ treatment, which were generally higher than those after 3 h GA_3_ treatment (Figure 6B). No obvious changes were noticed in the expression levels of *FaFAR1-4*, *FaFAR1-17*, *FaFAR1-21*, *FaFAR1-22*, *FaFAR1-28*, *FaFAR1-29*, *FaFAR1-33*, *FaFAR1-40*, *FaFAR1-45*, or *FaFAR1-47* genes after GA_3_ treatment, suggesting that these genes may not be involved in the GA_3_ signaling pathway.

Compared to CK, majority of *FaFAR1/FHY3* genes did not respond to the salicylic acid (SA) treatment. However, *FaFAR1-17*, *FaFAR1-28*, *FaFAR1-38*, and *FaFAR1-44* exhibited a remarkable upregulation, displaying strong SA-responsive features (Figure S5A). A quarter of the genes, including *FaFAR1-2*, *FaFAR1-5*, *FaFAR1-9*, *FaFAR1-15*, *FaFAR1-17*, *FaFAR1-20*, *FaFAR1-22*, *FaFAR1-28*, *FaFAR1-34*, *FaFAR1-38*, *FaFAR1-44*, and *FaFAR1-47*, showed significantly elevated expression under methyl jasmonate (MeJA) treatment (Figure S5B), indicating that these genes are actively involved in MeJA signaling.

Collectively, *FaFAR1-9* responded positively to ABA, GA_3_, and MeJA signaling and *FaFAR1-44* showed a high expression level after the SA and MeJA treatments. These results indicate that *FaFAR1-9* and *FaFAR1-44* could respond to multiple phytohormones. These findings provide crucial details to further explore the functions of *FaFAR1/FHY3* in phytohormone signaling in strawberries.

### 2.7. Responses of FaFAR1/FHY3 Genes to FR Light Signaling

**Figure 7:**
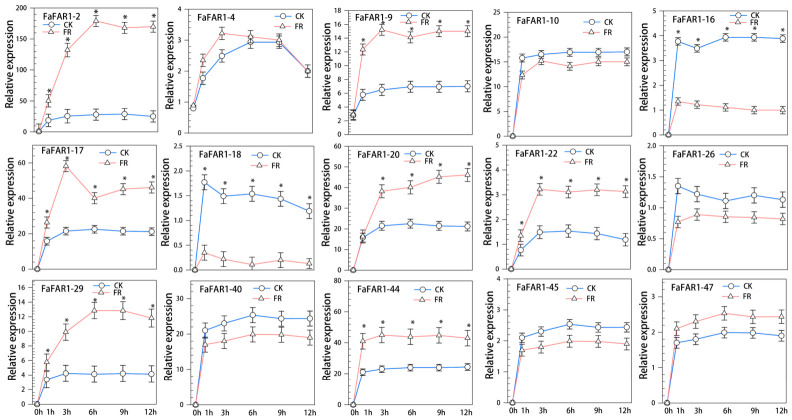
Expression patterns of *FaFAR1/FHY3* genes under far-red light treatment over 12 h. Significant differences are marked with an asterisk (*) at *p* < 0.05.

Most genes were rapidly upregulated under FR light conditions, peaking at 3–6 h, following gradual stabilization or slight decline, consistent with typical early FR light-responsive traits. Prominently enhanced expression of *FaFAR1-2*, *FaFAR1-9*, *FaFAR1-17*, *FaFAR1-20*, *FaFAR1-22*, *FaFAR1-29*, and *FaFAR1-44* was observed after FR light exposure (Figure 7). Expression levels of these genes were substantially higher than those under white light (CK), and persisted at a constantly high level under FR light conditions, highlighting the function of these genes in FR light signaling. The strongest response was noticed in *FaFAR1-2*, with a relative expression level of approximately 180 after 6 h FR light treatment (Figure 7). In contrast, *FaFAR1-16* and *FaFAR1-18* were evidently downregulated by FR light. The expression of *FaFAR1-10*, *FaFAR1-26*, *FaFAR1-40*, *FaFAR1-45*, and *FaFAR1-47* showed no significant differences compared to the CK, indicating their insensitivity to FR light. These results underline the distinct divergence of *FaFAR1/FHY3* gene responses to FR light.

### 2.8. Synergistic Action of the FaFAR1/FHY3 Genes in Anthocyanin Biosynthesis During Fruit Development

**Figure 8:**
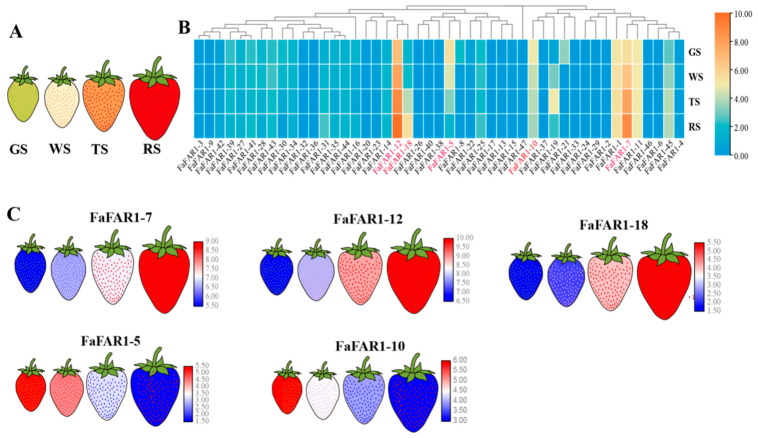
Expression atlas of *FaFAR1/FHY3* genes during strawberry fruit development. (**A**) Schematic illustration of strawberry fruit during four developmental stages. (**B**) Transcriptome-based expression heatmap of *FaFAR1* genes. Genes marked in red are the key ones to focus on. Results are from three biological replicates. Transcript abundance is normalized to transcripts per million (TPM) for gene expression analysis. (**C**) Visualization of relative expression levels of five key *FaFAR1* genes. GS: green stage; WS: white stage; TS: turning stage; RS: red stage.

Strawberry fruit development was divided into four stages—green stage (GS), white stage (WS), turning stage (TS), and red stage (RS)—based on fruit maturation (Figure 8A). The transcription profiles of the *FaFAR1/FHY3* genes during the four developmental stages of strawberry fruit indicated that most of them exhibited low or undetectable expression levels (Figure 8B). However, the transcript abundances of *FaFAR1-7*, *FaFAR1-12*, and *FaFAR1-18* gradually increased during fruit ripening, whereas opposite expression patterns were noticed in *FaFAR1-5* and *FaFAR1-10* (Figure 8B). The expression patterns of these five genes were also clearly visualized with a schematic heatmap of strawberry fruit at each developmental stage (Figure 8C). These results reveal the active participation of these five genes, which might play crucial roles in strawberry fruit development.

**Figure 9:**
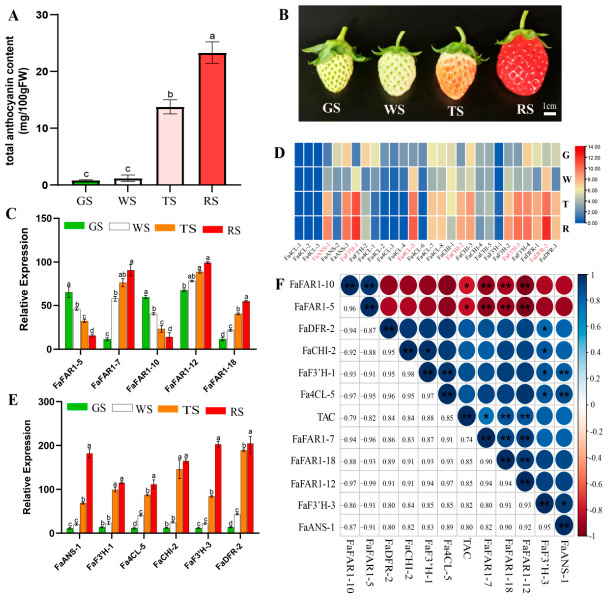
Total anthocyanin content and expression profiles of genes in anthocyanin biosynthesis pathway during strawberry development. (**A**) Total anthocyanin content. (**B**) Visual appearance of “Akihime” strawberry fruit during different developmental stages. Results are from three biological replicates. Transcript abundance is normalized to transcripts per million (TPM) for gene expression analysis. (**C**) qRT-PCR analysis of 5 key *FaFAR1/FHY3* genes during fruit development. (**D**) Expression heatmap of genes in anthocyanin biosynthesis during different stages of fruit development based on transcriptome data. Genes marked in red are the key ones to focus on. (**E**) qRT-PCR analysis of 6 key genes in anthocyanin biosynthesis during fruit development. (**F**) Correlation analysis of 5 key *FaFAR1/FHY3* genes, 6 key genes in anthocyanin biosynthesis pathway, and total anthocyanin content across stages GS-RS of strawberry fruit development. Blue and red represent positive and negative correlations, and larger circles represent stronger correlations. Values indicate correlation coefficients at 0.05 (*) and 0.01 (**) levels. GS: green stage; WS: white stage; TS: turning stage; RS: red stage. Different case letters in (**A**,**C**,**E**) indicate significant differences at 0.05 level.

To explore the potential roles of *FaFAR1/FHY3* genes in anthocyanin biosynthesis, the total anthocyanin content (TAC) was measured for strawberry fruits during the four developmental stages (Figure 9A,B). A significant increase in the TAC was noticed during fruit ripening (Figure 9A). A quantitative real-time PCR (qRT-PCR) was employed to measure the expression of the five genes (*FaFAR1-7*, *FaFAR1-12*, *FaFAR1-18*, *FaFAR1-5*, and *FaFAR1-10*) at each developmental stage, which indicated high consistency with the RNA-Seq data (Figure 8B,C and Figure 9C). Furthermore, both the transcriptomic profiles and qRT-PCR results revealed that the expression levels of another six key genes (*FaDFR-2*, *FaF3′H-1*, *FaF3′H-3*, *FaCHI-2*, *Fa4CL-5*, and *FaANS-1*), which participate in anthocyanin biosynthesis, also gradually increased during fruit ripening (Figure 9D,E).

A correlation heatmap of the five *FaFAR1/FHY3* genes, six key anthocyanin biosynthetic genes, and the TAC in strawberry fruit was constructed to dissect the regulatory relationships (Figure 9F). *FaFAR1-7*, *FaFAR1-12*, and *FaFAR1-18* showed significant positive correlations with the TAC and the six core anthocyanin biosynthetic genes during fruit development (r > 0.80), suggesting they might function as positive regulators in anthocyanin biosynthesis and accumulation. In contrast, *FaFAR1-5* and *FaFAR1-10* displayed strong negative correlations with the TAC and the anthocyanin biosynthetic genes (r = −0.79 to −0.97), indicating that these two genes might act as negative regulators during anthocyanin biosynthesis in strawberry fruit. Furthermore, significant positive correlations among the key anthocyanin biosynthetic genes (*FaDFR-2*, *FaCHI-2*, and *FaF3′H-1*) were also observed (Figure 9F), indicating that the co-expression of these genes promotes anthocyanin accumulation during strawberry fruit ripening.

### 2.9. Transcriptional Regulation of Anthocyanin Biosynthesis-Related Structural Gene Promoters by FaFAR1 Transcription Factors

**Figure 10:**
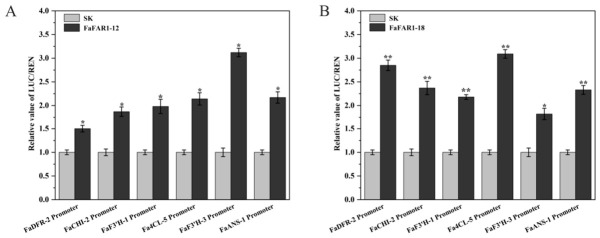
The regulatory effects of FaFAR1-12 (**A**) and FaFAR1-18 (**B**) on promoter of key structural genes involved in anthocyanin synthesis in strawberry fruits. Asterisk (*) and double asterisk (**) represent significant differences at *p* < 0.05 and *p* < 0.01, respectively.

A dual-luciferase reporter assay system was used to further verify the transcriptional regulatory effects of five FaFAR1 transcription factors (FaFAR1-5, FaFAR1-7, FaFAR1-10, FaFAR1-12 and FaFAR1-18) on the promoters of six key structural genes (*FaDFR-2*, *FaCHI-2*, *FaF3′H-1*, *Fa4CL-5*, *FaF3′H-3*, and *FaANS-1*) involved in strawberry fruit anthocyanin biosynthesis. The results show that FaFAR1-12 can activate the promoters of all six anthocyanin biosynthesis structural genes. Compared with the control, the relative transcriptional activities were 1.50-fold for *FaDFR-2*, 1.87-fold for *FaCHI-2*, 1.98-fold for *FaF3′H-1*, 2.14-fold for *Fa4CL-5*, 3.12-fold for *FaF3′H-3*, and 2.17-fold for *FaANS-1* (Figure 10A). In addition, FaFAR1-18 exhibited significant activating effects on the promoters of the six key anthocyanin biosynthesis structural genes. Its relative transcriptional activities were 2.85-fold for *FaDFR-2*, 2.37-fold for *FaCHI-2*, 2.18-fold for *FaF3′H-1*, 3.09-fold for *Fa4CL-5*, 1.82-fold for *FaF3′H-3*, and 2.33-fold for *FaANS-1* compared to those of the control (Figure 10B). By contrast, the other three FaFAR1 TFs (FaFAR1-5, FaFAR1-7 and FaFAR1-10) showed no obvious activation effects on the promoters of these six structural genes. Collectively, these findings indicate that FaFAR1-12 and FaFAR1-18 act as important regulators in strawberry fruit anthocyanin biosynthesis.

## 3. Discussion

### 3.1. Evolutionary Characteristics of FaFAR1/FHY3 Genes

A total of 47 *FaFAR1/FHY3* genes were verified in strawberry fruits, which were unevenly dispersed across 15 chromosomes (Figure S1). The quantity of *FaFAR1/FHY3* genes exceeded those in cucumber (20) [31], maize (16) [22], and *Eucalyptus* (33) [19]. Gene segmental duplication was a crucial driving force behind the expansion of genetic families during species evolution [32]. Here, 17 pairs of *FaFAR1/FHY3* genes were derived from segmental duplication events, accounting for 72.34% of all the *FaFAR1/FHY3* genes (Figure 1). This highlights gene duplication as a major contributor to the expansion of the *FaFAR1/FHY3* gene family. Similar results have also been documented in peanut [33] and walnut [24]. Furthermore, three tandemly duplicated gene clusters, encompassing eight genes in total, were found in *F. ananassa* (Figure S1), indicating that tandem duplication also contributed to the increase in *FaFAR1/FHY3* genes to some extent.

The phylogenetic analysis divided the *FaFAR1/FHY3* genes into six subfamilies (Figure 2A), and their distribution pattern was highly consistent with that of *A. thaliana* [16], reflecting the evolutionary conservation of the FAR1/FHY3 proteins in plants. Analysis of gene construction and motifs revealed that *FaFAR1/FHY3* display a distinct clade-specific pattern: genes within the same subfamily have identical or highly similar gene architectures and motif compositions (Figure 2B,C). The reliability of our subfamily classification of the *FaFAR1/FHY3* genes is validated by these findings. Moreover, phylogenetic analysis of 123 FAR1/FHY3 protein sequences from strawberry fruits and four other typical plant species (maize, tomatoes, *A. thaliana*, and *Pyrus bretschneideri*) divided them into seven groups. The largest number of *FaFAR1/FHY3* genes were found in Group II, while none were found in Group VII (Figure 3). Thus, it is speculated that the *FaFAR1/FHY3* genes belonging to Group VII might have been lost during the strawberry’s evolution, whereas an independent evolution might have been followed and unique biological functions might have been acquired by the *FaFAR1/FHY3* genes in Group II. The maize plant also reveals a similar evolutionary pattern [22].

The promoter prediction analysis indicated that the *FaFAR1/FHY3* genes were enriched in *cis*-acting elements associated with light, hormone, and stress responses, as well as with plant growth and development, which are consistent with the main results of early related research on *A. thaliana* [16], roses [34], and grapes [35]. The most abundant elements were LREs, and each *FaFAR1/FHY3* gene contained more than one LRE (Figure 4B). Moreover, Crosstalk might exist between light and temperature or phytohormone response pathways, as the promoter regions of most *FaFAR1/FHY3* genes contained conserved *cis*-acting elements, including Box4, G-box, low-temperature response element (LTRE), and ABA response element (ABRE).

### 3.2. Responses of FaFAR1/FHY3 Genes to Abiotic Stress and Hormone Treatments

Although the *FAR1/FHY3* genes were first characterized for their role in light signal transduction, recent studies have demonstrated that FAR1/FHY3 proteins can directly target multiple genes and widely participate in plant growth and development, including phytohormone signaling and biotic and abiotic stress responses [1,2,18,20]. It has also been reported that plant responses to salt stress are modulated by light, and the nuclear translocation of phyA and phyB is critical for this process [19,36,37,38]. Meanwhile, the AtHY5 and AtPIF4 proteins have been implicated in the salt tolerance pathway in *A. thaliana* by interacting with AtFHY3 and mediating the nuclear import of phytochromes [3,38]. Salt treatment significantly enhanced the expression levels of *FaFAR1-27* and *FaFAR1-41* (Figure 5A). A high evolutionary conservation of protein sequences among FaFAR1-27, FaFAR1-41, and AtFHY3 proteins was revealed by the phylogenetic analysis, signifying their highly similar functions (Figure 3). Did *FaFAR1-27* and *FaFAR1-41* regulate strawberry salt tolerance by modulating the nuclear translocation of phytochromes? Further experiments are required to verify these questions.

The expression of *FaFAR1-1*, *FaFAR1-7*, *FaFAR1-9*, and *FaFAR1-17* was significantly upregulated under low temperature (Figure 5B). Meanwhile, significantly elevated expression levels were displayed by four genes (*FaFAR1-7*, *FaFAR1-9*, *FaFAR1-30*, and *FaFAR1-43*) under high temperature (Figure 5C). The results indicate that *FaFAR1-7* and *FaFAR1-9* might be involved in both heat shock and low-temperature responses in strawberries. In *Eucalyptus grandis*, the *FAR1/FHY3* genes exhibit similar expression patterns in response to low and high temperatures, reflecting the conservation of these genes in mediating plant temperature stress responses [19]. Cold tolerance in tomatoes could be enhanced by *SlFHY3* [39]. Whether a similar regulatory mechanism is conserved in *FaFAR1-7* or *FaFAR1-9* remains to be confirmed. Low temperature prominently repressed the expression of nearly all the *FaFAR1/FHY3* genes (Figure 5B). This phenomenon may be associated with the overall reduction in gene expression under low-temperature conditions.

Excessive accumulation of ROS in plant cells is always induced by abiotic stresses, such as drought, high temperature, and salinity. Previous studies have demonstrated that ROS accumulation in *A. thaliana* is negatively regulated by *AtFHY3* and *AtFAR1* [7,12,40]. In this study, the consistent downregulation of *FaFAR1-40* under salt, temperature, and drought stresses raises the question of whether this gene is also involved in regulating ROS accumulation in strawberry plants under these abiotic stresses.

As key signaling molecules, plant hormones mediate plant adaptation to abiotic stresses [41,42]. The results reveal that both biotic and abiotic stresses effectively modulate the *FaFAR1/FHY3* genes’ expressions (Figure 5 and Figure S4), yet the molecular mechanisms by which the *FaFAR1/FHY3* genes regulate plant stress responses through hormone signaling pathways remain vague. In *A. thaliana*, the FHY3 and FAR1 proteins can directly bind to the promoter of the *abscisic acid-insensitive 5* (*ABI5*) gene, a critical component of the ABA signal-mediated stress response pathway [11]. In this study, *FaFAR1-9* was found to actively respond to both ABA signaling and abiotic stresses (Figure 5 and Figure 6A). Thus, it is hypothesized that *FaFAR1-9* might participate in plant stress responses by activating the ABA signaling pathway. Additionally, we observed that *FaFAR1-17*, *FaFAR1-28*, *FaFAR1-38*, and *FaFAR1-44* were significantly upregulated by the SA and MeJA treatments (Figure S4). Moreover, these genes were also strongly induced by abiotic or biotic stresses (Figure 5 and Figure S4). Similar results have been reported in potatoes [43]. Therefore, it is reasonable to speculate that stress-related phytohormones, such as SA and MeJA, might also be integrated into the plant’s stress response regulatory network by regulating the expression of the *FaFAR1/FHY3* genes in strawberries.

In conclusion, the *FaFAR1/FHY3* genes exhibited diverse expression patterns in response to abiotic and biotic stresses, as well as to phytohormone signaling, suggesting that these genes might be associated with distinct signal transduction pathways. Yet, the exact regulatory relationship and molecular mechanism linking SA/MeJA signaling to *FaFAR1/FHY3*-mediated stress responses remain unclear and require further systematic functional verification through genetic transformation, physiological phenotypic identification, and protein interaction experiments in future studies.

### 3.3. Responses of FaFAR1/FHY3 Genes to FR Light

The *AtFAR1* and *AtFHY3* genes acted as regulators of the phyA-mediated FR light response in *A. thaliana* [15]. The dynamic expression of the *FaFAR1/FHY3* genes in FR light-illuminated strawberry leaves was analyzed over 12 h. Seven genes (*FaFAR1-2*, *FaFAR1-9*, *FaFAR1-17*, *FaFAR1-20*, *FaFAR1-22*, *FaFAR1-29*, and *FaFAR1-44*) presented typical far-red light-responsive features (Figure 7), implying that these genes might be involved in the growth and development of strawberry plants under FR light conditions, while the remaining non-responsive genes might lack such functions. Similar findings have been observed in maize inflorescence tissues under FR light during different developmental stages [44].

### 3.4. Dynamic Expression of FaFAR1/FHY3 Genes and Identification of FaFAR1-12/18 as Core Positive Transcriptional Regulators in Anthocyanin Biosynthesis During Strawberry Fruit Development

This study found that the expression of *FaFAR1-12* and *FaFAR1-18* during the different developmental stages of strawberry fruits showed an extremely significant positive correlation with the fruit’s TAC and the expression of six key genes in anthocyanin biosynthesis (*FaDFR-2*, *FaCHI-2*, *FaF3′H-1*, *Fa4CL-5*, and *FaANS-1*) (Figure 9). Considering the limited sample size, with only three biological replicates for the correlation analysis, we further performed dual-luciferase reporter assays to validate our findings. The dual-luciferase reporter assays also revealed that FaFAR1-12 and FaFAR1-18 significantly activated the promoters of key structural genes involved in anthocyanin biosynthesis (Figure 10). These results suggest that *FaFAR1-12* and *FaFAR1-18* TFs might play crucial regulatory roles in anthocyanin synthesis during strawberry fruit development. However, the lack of in planta functional identification is an important limitation of the present study. In addition, genotype differences among various cultivars may have introduce confounding factors into the experimental results and follow-up experiments will focus on one fixed strawberry cultivar to systematically verify our research findings.

Both *fhy3* and *far1* mutants exhibited an anthocyanin accumulation-deficient phenotype in *A. thaliana*, characterized by a significantly lower anthocyanin content in mutant seedlings under FR or white light [45,46]. In the light-regulated anthocyanin accumulation pathway, the *FAR1/FHY3* TFs were important upstream components in *A. thaliana*, and their core function was to indirectly activate the downstream anthocyanin biosynthetic pathway by phyA signal homeostasis, which confirmed that anthocyanin accumulation in *A. thaliana* was positively regulated by *FAR1/FHY3* [16,46]. Anthocyanin accumulation regulated by FAR1/FHY3 in *Arabidopsis* mainly occurred in seedlings, whereas in octoploid strawberry fruits, FaFAR1 TF-mediated anthocyanin biosynthesis was predominantly fruit specific. We systematically compared the conserved regulatory functions and divergent tissue-specific features of *FAR1/FHY3* homologs between the model plant *Arabidopsis* and fleshy-fruited strawberry plants, and propose that such interspecific differences serve as an important contributor to the evolutionary divergence in the tissue location of anthocyanin biosynthesis. In *Paeonia rockii*, the PrFRS2 protein can activate the expression of *PrMYB75a* and *PrANS* by binding to the *FAR1/FHY3* promoter, promoting anthocyanin accumulation in the petals [47]. However, the presence of such mechanisms in the anthocyanin biosynthetic pathway of strawberry fruits remains to be explored further.

Combined with our fruit phenotypic data, qRT-PCR detection results and dual-luciferase reporter assay evidence, we preliminarily speculate that FaFAR1-12 and FaFAR1-18 separately target the key structural genes in the anthocyanin biosynthesis pathway of strawberry fruits. They strongly activate the transcription of these downstream structural genes, significantly elevate anthocyanin accumulation, and thereby positively regulate anthocyanin synthesis in strawberry fruits. Nevertheless, this proposed regulatory model remains tentative and needs in-depth experimental verification in future research.

Under high temperature, *VvFHY3* participated in the regulation of the “VvARF3-VvFHY3-VvbZIP17” module, which jointly inhibited the expression and synthesis of anthocyanin structural genes, negatively modulating anthocyanin accumulation in grape fruits [48]. Although the expression of *FaFAR1-5* and *FaFAR1-10* presented highly negative correlations with the fruit’s TAC and the expression of key structural genes in anthocyanin biosynthesis (*FaDFR-2*, *FaCHI-2*, *FaF3′H-1*, *Fa4CL-5*, and *FaANS-1*) during strawberry fruit development (Figure 9), FaFAR1-5 and FaFAR1-10 could not effectively activate the promoters of these six key structural genes (Figure S6), implying that these two TFs do not exert an obvious regulatory effect on anthocyanin biosynthesis in strawberry fruits. It is possible that these TFs have an evolved functional divergence in strawberry fruits.

## 4. Materials and Methods

### 4.1. Plant Materials

Four-week-old “Yoshino” strawberry seedlings were transplanted into 0.3 L cylindrical plastic pots containing 0.25 L medium (perlite, vermiculite, and peat with a volume ratio of 1:1:2). Seedlings were cultured in growth chamber (photoperiod: 14 h light/10 h dark; temperature: 23 ± 2 °C/15 ± 2 °C day/night; relative humidity (RH): 75 ± 5%; LED white light at an intensity of 200 μmol·m^−2^·s^−1^). Hoagland nutrient solution was used to manually irrigate plantlets 2–3 times weekly. Seedlings were subjected to subsequent treatments after two weeks of acclimatization. Unless otherwise specified, same growing conditions were utilized for strawberry seedlings throughout study. Given distinct varietal sensitivity to diverse treatments, multiple octoploid cultivated strawberry cultivars were selected to systematically characterize expression responsiveness of *FaFAR1/FHY3* family genes across different genetic backgrounds. All strawberry materials used in this study belong to octoploid commercial *Fragaria* × *ananassa* (2n = 8x = 56), and detailed cultivar information is described in corresponding experimental sections.

Transcriptome data of strawberry fruits at different developmental stages were retrieved from European Nucleotide Archive (https://www.ebi.ac.uk/ena, accessed on 30 January 2025) under accession number PRJEB12420 [49]. Fruits of “Camarosa” were collected at GS, WS, TS, and RS for RNA sequencing. Healthy fruits with similar shape, size, and color at each stage were harvested simultaneously and immediately transported to laboratory on ice, rapidly frozen in liquid N_2_, and finally stored at −80 °C. Raw RNA-seq reads were mapped to strawberry reference genome using HISAT2 v2.2.1 as alignment tool. Gene expression quantification was calculated with StringTie v2.2.1, and transcript abundance was finally normalized to TPM (transcripts per million) for subsequent expression analysis.

For qRT-PCR and fruit TAC determination, strawberry fruit samples of “Akihime” cultivar at four developmental stages were purchased from commercial farm (CiyiJia Farm, Ningbo, China). Fruit selection, harvesting, pre-treatment, and storage procedures were same as mentioned above.

### 4.2. Identification and Analysis of FaFAR1/FHY3 Genes

The genome data of *F. ananassa* were downloaded from the strawberry genome website (http://www.strawberrygenome.org, accessed on 30 January 2025). The reference gene sequences for *AtFAR1/FHY3* were obtained from the *A. thaliana* website. The three typical conserved domains of FAR1/FHY3 family are FAR1, MULE and SWIM, corresponding to the PFAM (https://www.ebi.ac.uk/interpro/, accessed on 30 January 2025) accession numbers PF03101, PF10551 and PF04434, respectively. The hidden Markov model was downloaded with the standard PFAM accession numbers, and the E-value cutoff was set to 1 × 10^−5^ during genome-wide gene screening. The HMM file of the FAR1/FHY3 protein domains obtained from the InterPro database (https://www.ebi.ac.uk/interpro/, accessed on 30 January 2025) were used as a seed file to scan the target protein sequences in the strawberry fruits. The conserved domains were manually verified by submitting the protein sequences to the SMART database (https://smart.embl.de, accessed on 30 January 2025) and the NCBI Conserved Domain Database (https://www.ncbi.nlm.nih.gov/cdd/, accessed on 30 January 2025).

Expasy (https://www.expasy.org, accessed on 30 January 2025) was used to analyze the physicochemical properties of the FaFAR1/FHY3 proteins. Their subcellular localization was performed through Euk-mPLoc 2.0. The gene annotation file for *F. ananassa* and the MEME website were used to analyze the gene structure, chromosome localization, and motif analysis; TBtools-II v2.441 was used to visualize the results.

### 4.3. Evolutionary Analysis of FAR1/FHY3 Proteins

A multiple sequence alignment with ClustalX v2.1 was performed for the 123 *FAR1/FHY3* protein sequences from *F. ananassa*, *S. lycopersicum*, *A. thaliana*, *O. sativa*, and *Pyrus bretschneideri* Rehd. The best-fit amino acid substitution model was LG + G +I, and a bootstrap analysis with 1000 replicate tests was performed to evaluate the reliability of each branching node. A phylogenetic tree was constructed by MEGA7.0 with the maximum likelihood method, and FigureTree v1.4.4 software was used to visualize the results.

### 4.4. Synteny Analysis and Promoter Cis-Acting Elements Prediction

The intraspecific synteny analysis of the *FaFAR1/FHY3* genes was conducted using TBtools. The genome data of *S. lycopersicum*, *A. thaliana*, *O. sativa*, *P. communis*, *R. chinensis*, *P. persica*, *Z. mays*, and *R. fruticosus* were retrieved from the Ensembl Plants website (https://plants.ensembl.org, accessed on 30 January 2025). The interspecific synteny analysis was performed between the strawberry and the other plant species. The *cis*-acting elements in the promoter were analyzed via the PlantCARE website (https://bioinformatics.psb.ugent.be/webtools/plantcare/html/, accessed on 30 January 2025) for 2000 bp upstream sequences of *FaFAR1/FHY3* genes.

### 4.5. Phytohormone Treatment

Leaves from the “Yoshino” plantlets were sampled at 3 h and 12 h after application of 100 μM ABA and gibberellic acid (GA_3_) to the six-week-old strawberry plantlets. An equal amount of ddH_2_O was applied to the control plantlets. The SA treatment data were retrieved from the Genome Sequence Archive in the BIG Data Center (https://ngdc.cncb.ac.cn/gsa, accessed on 30 January 2025) under accession numbers CRA001964 with a ‘Camarosa’ cultivar [50]. Two groups of seedlings were cultured in a greenhouse: one was treated with ddH_2_O and the other was treated with SA; leaf samples were collected for RNA-Seq 3 days later.

The MeJA treatment data for the transcriptome are under the accession number PRJNA508389 [51]. The plant samples (leaves treated with MeJA 24 h later) were collected from a “Camarosa” cultivar grown in a growth chamber.

### 4.6. Multiple Stresses Treatment

Salt stress was simulated using a 50 mol/L NaCl solution, while an equal amount of deionized water was used for the control group. Fully expanded functional leaves from the “Yoshino” plantlets were sampled for transcriptome sequencing after 7 days of treatment. The accession number of raw sequence data is SRA065786 [52].

Cold-treatment transcriptomic data from the GEO database (accession number GSE73488) were used [53]. Under 90 mol m^−2^ s^−1^ light illumination and 80% RH, strawberry plantlets were transferred to a culture room at 2 °C. Functional leaves from “Korona” plantlets were sampled 24 h and 72 h after cold initiation. Control samples (0 h) were harvested before.

The heat treatment data were retrieved from the NCBI Sequence Read Archive (SRA) database under accession number PRJNA1188098 [54]. High-temperature-tolerant “Portola” and high-temperature-sensitive “Fronteras” were selected. Following 6 days of treatment, the leaf samples were collected from the heat-treated (40 °C) and control (25 °C) plantlets.

The drought treatment data were obtained from the SRA database (BioProject ID PRJNA1366720) [55]. Based on the field capacity (FC), the drought levels were established with 75% FC as the control (CK), 55% FC as mild drought, and 45% FC as severe drought. Following 0, 6, and 18 days of drought treatment, fully expanded leaf samples from “Akihime” strawberry plantlets were used for the RNA-Seq. This study used sequencing data obtained after 6 days of treatment.

The transcriptome data of inoculation with *Xanthomonas fragariae* were obtained from the GEO database (accession number GSE150636) [56]. Strawberry plantlets, cultivar “Elsanta”, were inoculated by spraying *X. fragariae* onto the foliage. Both the control and inoculated plantlets were placed in a growth chamber at 80% RH within 30 days after pathogen inoculation to facilitate pathogen infection, with a 16 h light period at 22 °C and an 8 h dark period at 17 °C. Leaf samples were obtained at days 12 and 19 dpi for RNA extraction.

The transcriptome data for strawberry plants infected with *Colletotrichum acutatum* were obtained from the GEO database (accession number GSE56296) [57]. The *Colletotrichum acutatum* pathogen was used to inoculate eight-week-old cultivar “Camarosa” plantlets. Isolated *C. acutatum* CECT 20240 conidia were suspended in sterile water, with 10^4^ CFU mL^−1^ spraying on the strawberry plantlets. The control group was sprayed with sterile water only. The crowns of plantlets were sampled at 24 h post-inoculation for transcriptome sequencing.

### 4.7. FR Light Treatment

FR light treatment with 75 μmol·m^−2^·s^−1^ light intensity was applied to six-week-old strawberry seedlings. Leaf samples were collected at 0, 1, 3, 6, 9, and 12 h after the FR light treatment. The same intensity of white light was used for the control seedlings. The strawberry plant cultivar “Yoshino” was used in this experiment.

### 4.8. qRT-PCR

The total RNA of about 0.1 g of the frozen samples from the “Akihime” cultivar was extracted using an RNA kit (Vazyme Biotech Co., Ltd., Nanjing, China). All the experiments were performed with three independent biological replicates, and each biological replicate included three technical replicates to guarantee the reproducibility and accuracy of the quantitative results. The purified RNA was strictly assessed for integrity, purity, and concentration through agarose gel electrophoresis and spectrophotometry. The purified RNA was reverse-transcribed into cDNA after quality control. The TaqMan (Vazyme Biotech Co., Ltd., Nanjing, China) probe method was used to perform the qRT-PCR, with *FaActin* as the internal reference gene. All primer sequences, annealing temperatures, and predicted amplicon sizes for the target genes are listed in Table S3. The relative gene expression levels were calculated by the 2^−ΔΔCt^ method based on the threshold cycle (Ct) values of target genes and the internal reference gene [58]. All expression data are presented as the mean ± standard deviation (SD) of three biological replicates.

### 4.9. Anthocyanin Content Determination

The strawberry fruit samples from the “Akihime” cultivar were extracted with 1% (*v*/*v*) hydrochloric acid–methanol solution, which was pre-cooled in the dark for 12 h. After centrifugation at 4 °C with 12,000 r/min for 15 min, the supernatant was collected, and the TAC was determined using the pH differential method [59]. The TAC was expressed as mg per 100 g fresh weight (FW), using cyanidin-3-glucoside as a standard. All experiments were performed with three independent biological replicates, and each biological replicate included three technical replicates.

### 4.10. Dual-Luciferase Reporter Assay

The full-length open reading frames (ORFs) of FaFAR1-5, FaFAR1-7, FaFAR1-10, FaFAR1-12 and FaFAR1-18 were amplified and inserted into the pGreenII 0029 62-SK vector to construct transcription factor recombinant plasmids. The promoter sequences of six anthocyanin biosynthesis-related structural genes (*FaDFR-2*, *FaCHI-2*, *FaF3′H-1*, *Fa4CL-5*, *FaF3′H-3*, and *FaANS-1*) were separately cloned into a pGreenII 0800-LUC reporter vector. All recombinant plasmids were electrotransformed into the Agrobacterium tumefaciens strain GV3101. The primer sequences used for vector construction are listed in Table S4.

The agrobacterial strains carrying SK-transcription factor recombinant plasmids and LUC-promoter recombinant plasmids were streaked on LB solid medium supplemented with 50 μg/mL kanamycin (Kan) and 25 μg/mL gentamicin (Gen), respectively. After incubation at 28 °C for 2 days, single colonies were transferred to fresh resistant LB medium for overnight incubation to activate the strains. The activated agrobacterial cells were collected and resuspended in infiltration buffer (10 mM/L MES, 10 mM/L MgCl, 150 μM/L acetosyringone, pH 5.6), and the bacterial suspension density was adjusted to an OD of 0.75. Subsequently, the agrobacterial suspensions of transcription factors and promoters were mixed at a volume ratio of 10:1. For agroinfiltration, three healthy leaves of each Nicotiana benthamiana plant were injected with the mixed bacterial suspension. The combination of empty pGreenII 0029 62-SK vector and each structural gene promoter–LUC plasmid was used as the negative control.

All tobacco plants were cultivated in an artificial climate chamber with a 16 h/8 h light/dark cycle, a light intensity of 300 μmol/(m·s), a constant temperature of 25 °C, and a relative humidity of 50–75%. Three days after infiltration, leaf discs with a diameter of 2 mm were punched near the injection sites, with 1–2 leaf discs collected per leaf. The collected leaf discs were homogenized in 100 μL of 1× PBS buffer, and 50 μL of the supernatant was taken for enzymatic activity detection. The dual-luciferase activity was determined using a Modulus Luminometer (Promega, Madison, WI, USA) in accordance with the manufacturer’s instructions for the Dual-Luciferase Reporter Assay System. The transcriptional regulatory effect of transcription factors on the target promoters was evaluated by the LUC/REN ratio. The LUC/REN value of the negative control group (empty SK vector combined with each structural gene promoter) was defined as 1.0 to calculate the relative regulatory activity of each FaFAR1 TF. Each experimental group contained three biological replicates, and all experiments were independently repeated three times to verify the regulatory relationship between the transcription factors and target gene promoters. The “Akihime” cultivar was used in this experiment.

For each effector–reporter combination in the dual-luciferase assay, three independent biological replicates of *Agrobacterium* infiltration on the tobacco leaves, and three technical replicates were measured for each biological sample. The leaf tissues were harvested 48 h after agroinfiltration; the infiltrated leaf discs were excised, rapidly frozen in liquid nitrogen and stored at −80 °C before protein extraction. The LUC/REN ratio was normalized against the corresponding empty vector control as the baseline, and all data normalization strictly relied on the REN internal reference values. The statistical analysis was carried out using an independent sample *t*-test for pairwise comparisons between the control and treatment groups.

### 4.11. Statistical Analysis

GraphPad Prism 10.12 was used to perform the statistical analysis and generate the bar charts and line graphs. Gene expression heatmaps and cartoon heatmaps were constructed using TBtools. A Pearson correlation was adopted in this work and the correlation coefficient and significance are represented in the correlation heatmaps, which were generated in R software v4.6.0.

## 5. Conclusions

This study systematically identified 47 *FaFAR1/FHY3* genes in the *F. ananassa* genome. The phylogenetic analysis grouped these genes into six subfamilies, and gene structure and motif composition analyses revealed the evolutionary differences in the *FaFAR1/FHY3* genes. Gene duplication was the major driving force behind the expansion of these genes. The promoter and gene expression analyses revealed that the *FaFAR1/FHY3* genes actively responded to FR light, phytohormones, and abiotic and biotic stresses, with multiple genes exhibiting simultaneous responsiveness to various environmental signals. For instance, *FaFAR1-7* and *FaFAR1-9* were responsive to both high- and low-temperature stress; *FaFAR1-7* also mediated the drought stress response; *FaFAR1-3* was involved in both salt and drought stress responses. This multi-responsiveness suggests that *FaFAR1/FHY3* might be multifunctional in regulating distinct plant signal transduction pathways. The expression patterns of *FaFAR1-7*, *FaFAR1-12*, and *FaFAR1-18* during the different development stages of strawberry fruit were highly positively correlated with the fruit anthocyanin content and the expression of key structural genes involved in the anthocyanin biosynthetic pathway, suggesting their important roles in regulating anthocyanin synthesis in strawberry fruits. This study revealed the responses of the *FaFAR1/FHY3* genes to multiple stressors. Additionally, valuable candidate genes for genetic improvement of strawberry fruit quality were also screened.

## Figures and Tables

**Table 1:** Physicochemical properties of FaFAR1/FHY3 proteins.

Gene Name	Length (aa)	MW (KDa)	pI	Instability Index	GRAVY	Subcellular Localization
FaFAR1-1	819	93.44	8.42	39.21	−0.484	Nucleus
FaFAR1-2	795	92.16	8.45	45.44	−0.585	Nucleus
FaFAR1-3	901	103.00	7.24	52.22	−0.611	Nucleus
FaFAR1-4	941	106.53	8.21	39.46	−0.617	Nucleus
FaFAR1-5	691	80.33	5.94	45.24	−0.359	Nucleus
FaFAR1-6	792	89.84	8.76	43.42	−0.689	Nucleus
FaFAR1-7	563	65.72	7.1	38.98	−0.544	Nucleus
FaFAR1-8	691	80.31	5.88	45.51	−0.375	Nucleus
FaFAR1-9	897	102.83	7.62	53.15	−0.635	Nucleus
FaFAR1-10	806	93.12	8.23	48.49	−0.482	Nucleus
FaFAR1-11	761	88.15	7.31	42.18	−0.607	Nucleus
FaFAR1-12	835	96.21	6.48	52.68	−0.505	Nucleus
FaFAR1-13	737	84.18	6.57	49.31	−0.349	Nucleus
FaFAR1-14	835	96.24	6.59	52.06	−0.526	Nucleus
FaFAR1-15	737	84.15	7.01	50.38	−0.363	Nucleus
FaFAR1-16	578	67.08	9.09	48.95	−0.997	Nucleus
FaFAR1-17	732	83.81	6.85	49.14	−0.373	Nucleus
FaFAR1-18	835	96.37	6.76	51.42	−0.53	Nucleus
FaFAR1-19	576	67.00	9.02	43.52	−0.481	Nucleus
FaFAR1-20	524	59.10	6.35	50.6	−0.455	Nucleus
FaFAR1-21	691	78.81	8.95	41.5	−0.594	Nucleus
FaFAR1-22	640	73.58	8.93	46.55	−0.265	Nucleus
FaFAR1-23	631	72.20	6	51.96	−0.423	Nucleus
FaFAR1-24	910	103.85	6.99	48.66	−0.569	Nucleus
FaFAR1-25	679	77.85	8.49	49.52	−0.332	Nucleus
FaFAR1-26	690	79.52	5.97	43.41	−0.32	Nucleus
FaFAR1-27	866	98.94	5.87	51.59	−0.655	Nucleus
FaFAR1-28	896	101.79	6.07	45.37	−0.528	Nucleus
FaFAR1-29	899	101.78	6.91	39.77	−0.587	Nucleus
FaFAR1-30	668	76.78	5.6	43.88	−0.394	Nucleus
FaFAR1-31	669	77.17	8.43	42.04	−0.404	Nucleus
FaFAR1-32	744	86.60	5.26	45.78	−0.614	Nucleus
FaFAR1-33	688	79.27	8.69	42.29	−0.751	Nucleus
FaFAR1-34	641	74.02	6.46	44.09	−0.362	Nucleus
FaFAR1-35	669	77.19	8.34	44.89	−0.402	Nucleus
FaFAR1-36	703	81.57	5.28	45.81	−0.581	Nucleus
FaFAR1-37	742	86.13	9.1	40.49	−0.538	Nucleus
FaFAR1-38	691	79.79	6.09	42.28	−0.323	Nucleus
FaFAR1-39	863	98.56	5.97	50.66	−0.64	Nucleus
FaFAR1-40	690	79.37	5.94	41.31	−0.302	Nucleus
FaFAR1-41	848	97.04	5.95	53.32	−0.657	Nucleus
FaFAR1-42	835	96.06	6.46	52.64	−0.57	Nucleus
FaFAR1-43	896	101.84	6.05	46.59	−0.53	Nucleus
FaFAR1-44	669	77.21	8.24	43.36	−0.402	Nucleus
FaFAR1-45	786	87.97	8.56	39.63	−0.7	Nucleus
FaFAR1-46	866	97.94	8.49	42.18	−0.713	Nucleus
FaFAR1-47	606	69.04	8.72	50.42	−0.793	Nucleus

Note: MW: molecular weight; GRAVY: grand average of hydropathicity.

## Data Availability

The original contributions presented in this study are included in the article/Supplementary Materials. Further inquiries can be directed to the corresponding author.

## Supplementary Materials

The following supporting information can be downloaded at: https://www.mdpi.com/article/10.3390/ijms27125479/s1.
