# Supplementary material for: Genome-Wide Analysis Reveals the Roles of FAR1/FHY3 Genes in Fragaria × ananassa Under Abiotic/Biotic Stresses and Highlights Their Potential Functions in Anthocyanin Biosynthesis During Fruit Development
Source: Int J Mol Sci. 2026 Jun 17;27(12):5479. doi: 10.3390/ijms27125479 (PMC13299393; doi:10.3390/ijms27125479)
Supplement: Supplementary file 1 [file ijms-27-05479-s001.zip › Supplementary Figures20260606.docx]

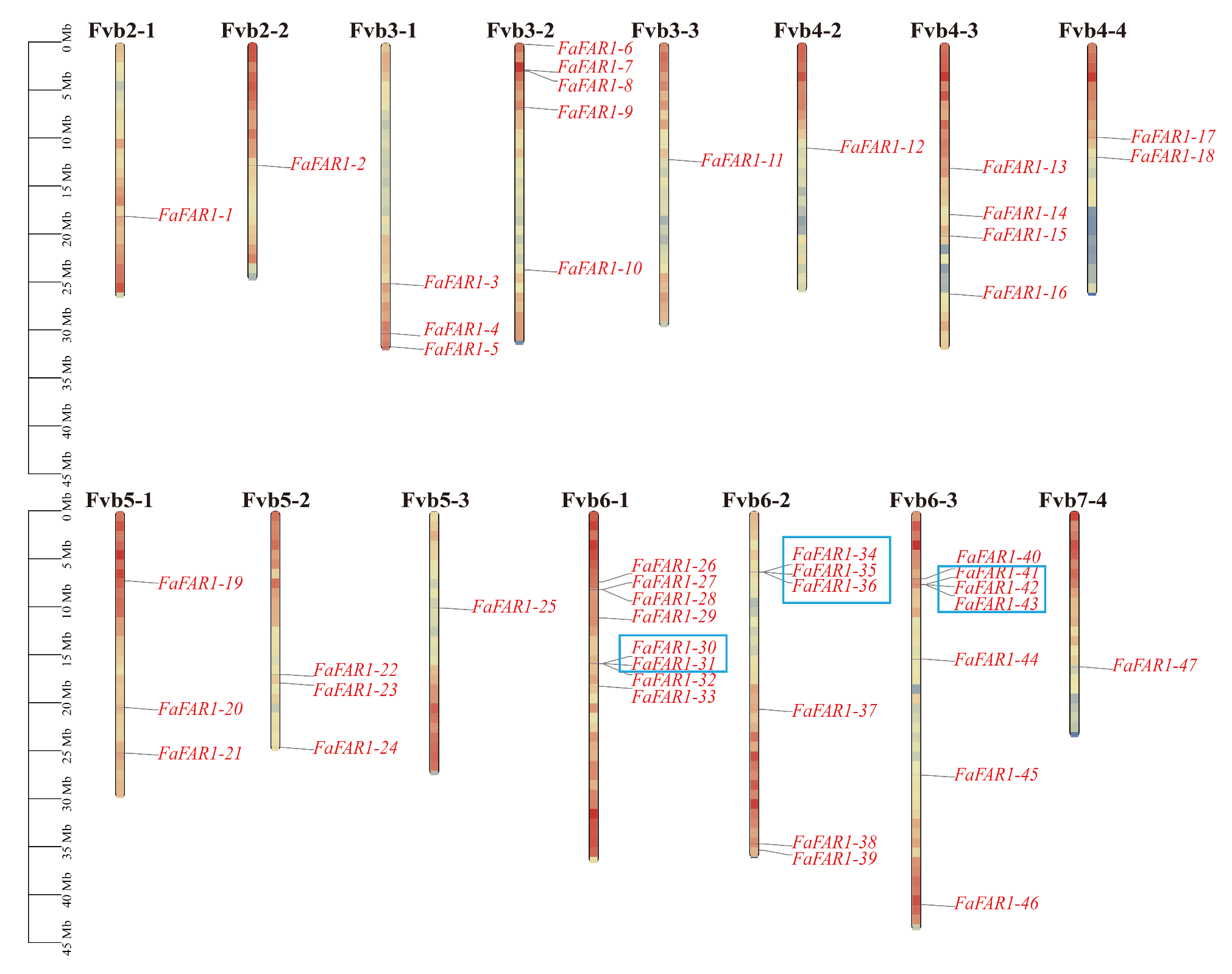


Figure S1. Chromosomal localization of FaFAR1/FHY3. Blue boxes indicate tandemly duplicated gene clusters.


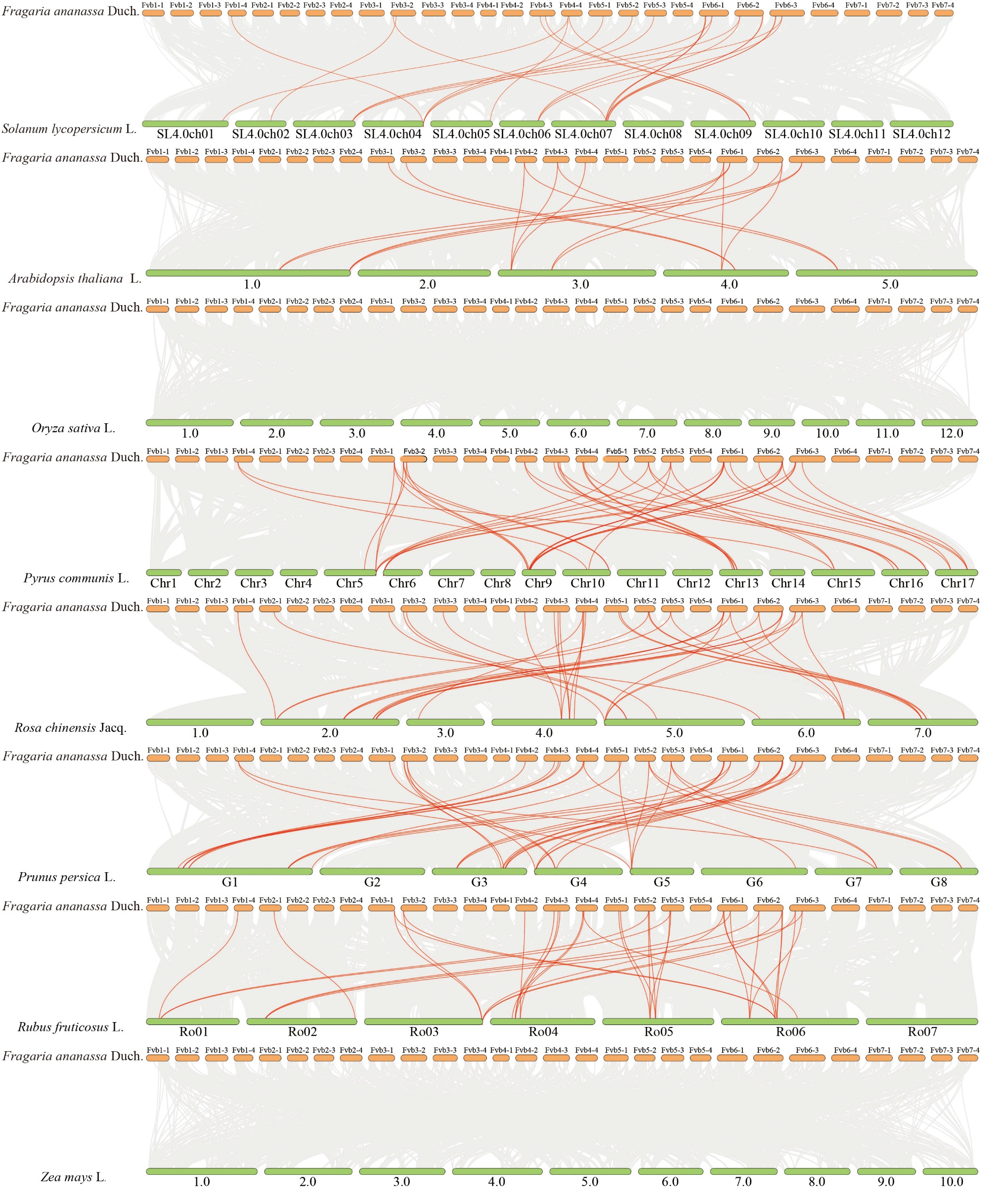


Figure S2. Collinearity analysis of FaFAR1/FHY3 genes between strawberry and other other representative plant species.


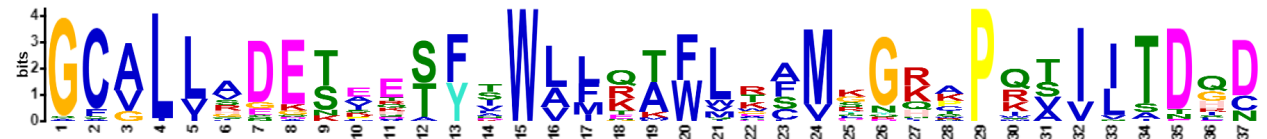

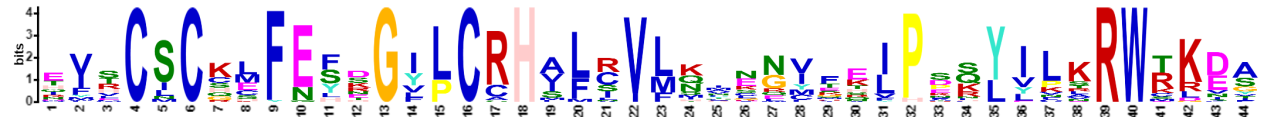

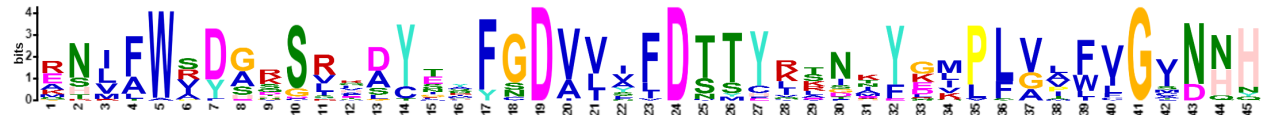

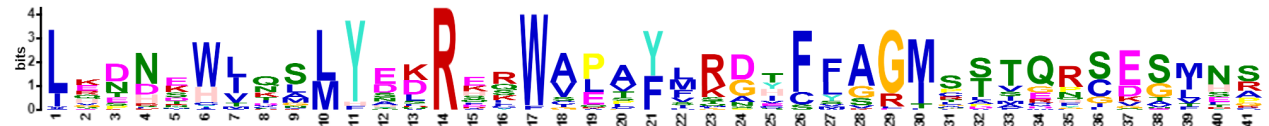

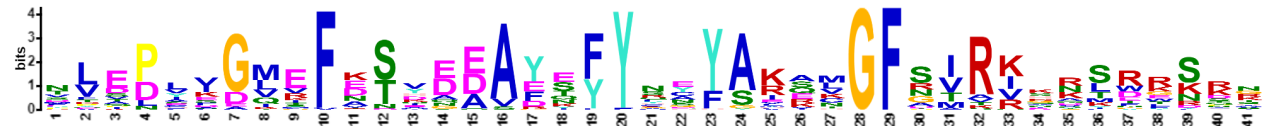

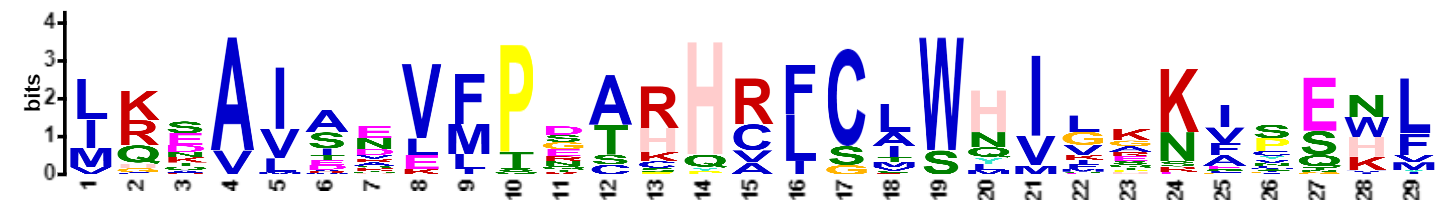

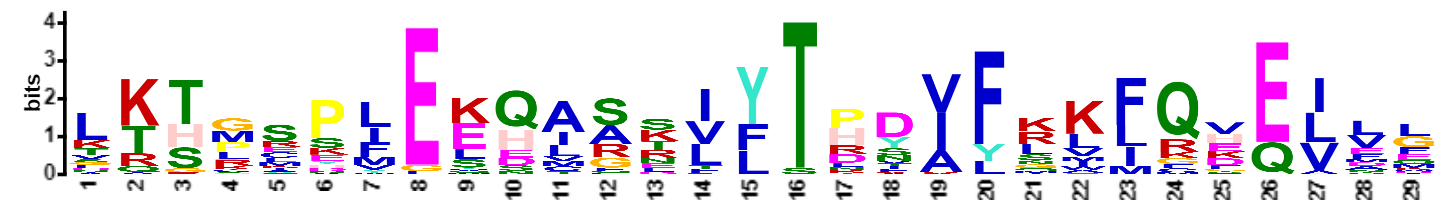

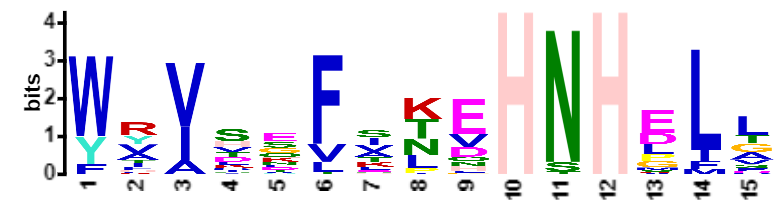

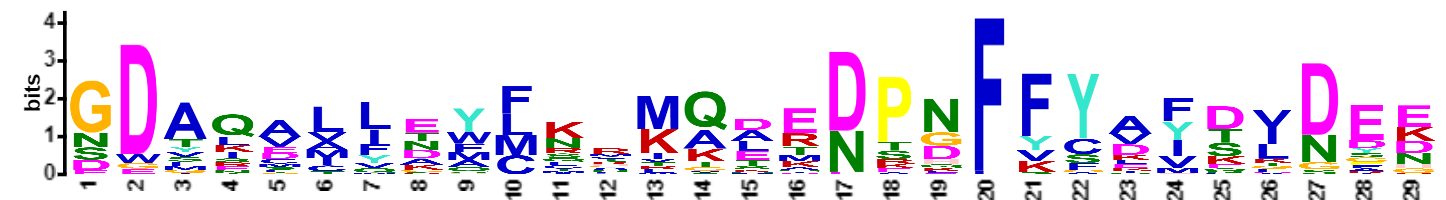

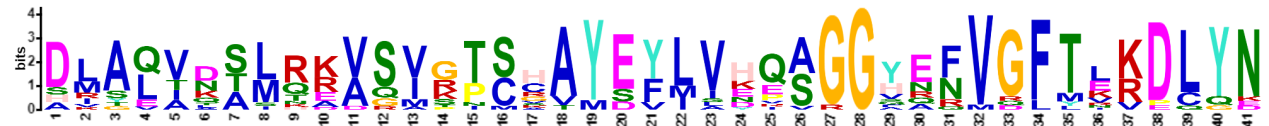


Motif 1

Motif 2

Motif 3

Motif 4

Motif 5

Motif 6

Motif 7

Motif 8

Motif 9

Motif 10

Figure S3. The Motif logo of FaFAR1/FHY3 genes.


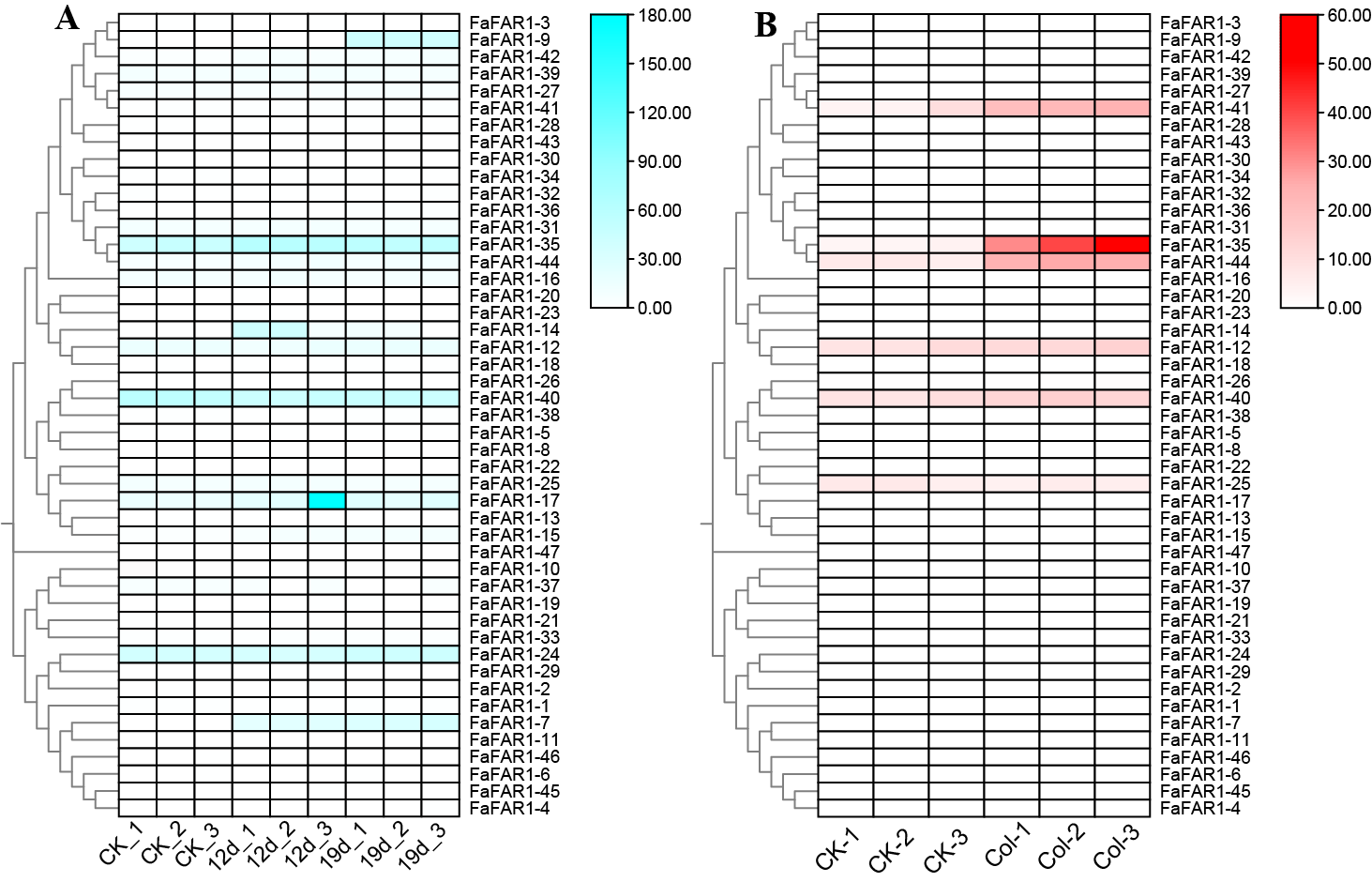


Figure S4. Expression analysis of FaFAR1/FHY3 under biotic stress. (A) Expression patterns of the *FaFAR1/FHY3* genes at 12 and 19 days post-inoculation with *Xanthomonas fragariae*. (B) Expression patterns of the *FaFAR1/FHY3* genes after inoculation with *Colletotrichum acutatum*.

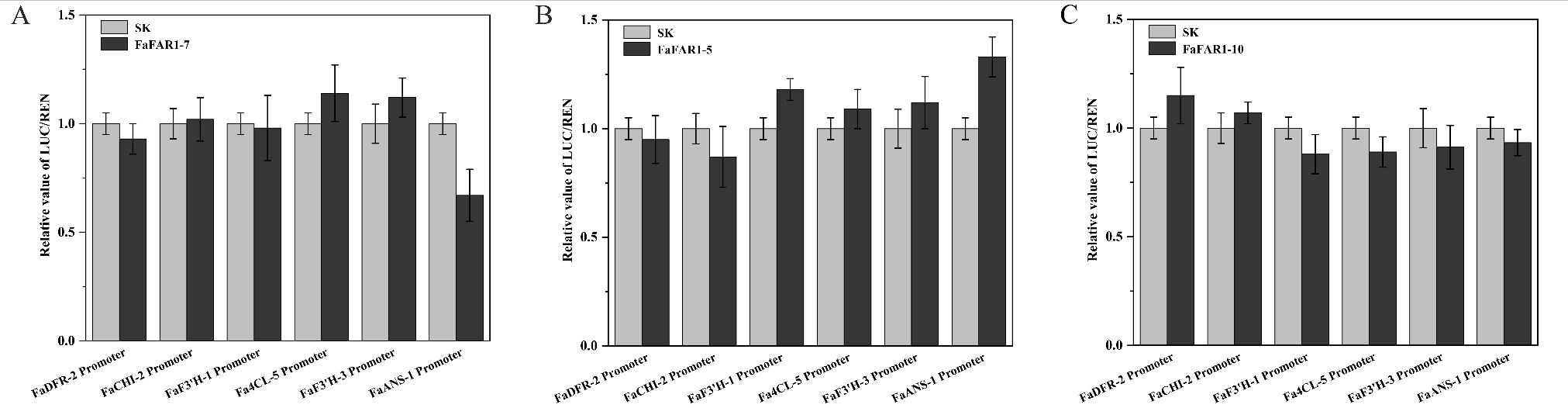


Figure S5. The regulatory effects of FaFAR1-7 (A), FaFAR1-5 (B) and FaFAR1-10 (C) on the promoter of key structural genes involved in anthocyanin synthesis in strawberry fruits. Asterisk (*) and double asterisk (**) represent significant differences at P < 0.05 and P < 0.01, respectively.

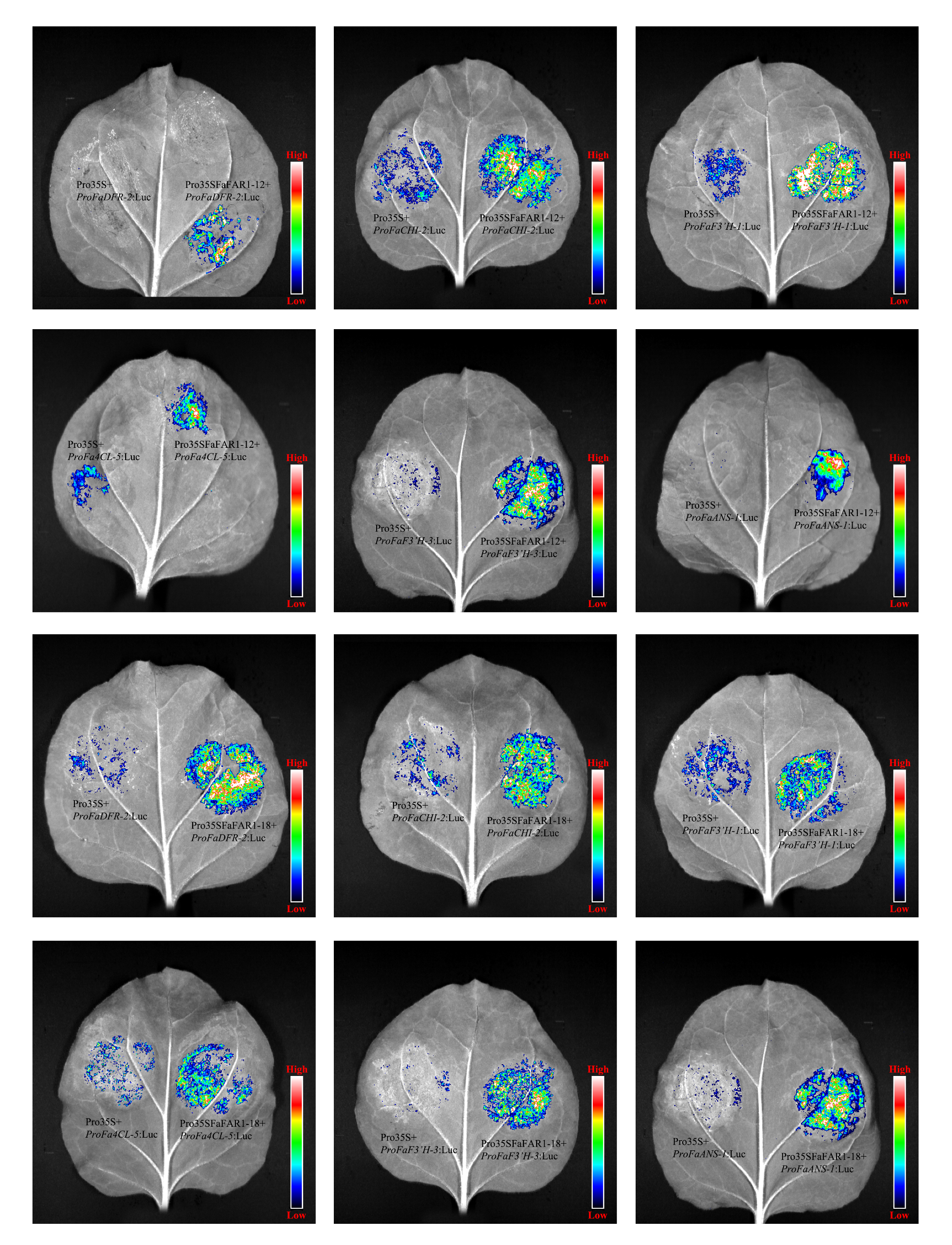


Figure S6. In vivo luminescence imaging for FaFAR1-12/18mediated activation of *FaDFR*, *FaCHI*, *FaF3H* and *FaANS* promoters via dual-luciferase assay in tobacco leaves.
